# Pelagic amphipods (Crustacea, Amphipoda, Hyperiidea) from the southern Gulf of Mexico with notes on the distribution of species

**DOI:** 10.3897/BDJ.11.e97347

**Published:** 2023-01-30

**Authors:** Laura Sanvicente-Añorve, Barbara Velázquez Ramírez, Margarita Hermoso-Salazar

**Affiliations:** 1 Instituto de Ciencias del Mar y Limnología, Universidad Nacional Autónoma de México, Mexico City, Mexico Instituto de Ciencias del Mar y Limnología, Universidad Nacional Autónoma de México Mexico City Mexico; 2 Facultad de Ciencias, Universidad Nacional Autónoma de México, Mexico City, Mexico Facultad de Ciencias, Universidad Nacional Autónoma de México Mexico City Mexico

**Keywords:** zooplankton, planktonic amphipods, hyperiids, epipelagic zone, dataset, occurrence, Gulf of Mexico

## Abstract

**Background:**

Studies referring the amphipod diversity have been mainly focused on the benthic environment. This study aimed to analye the epipelagic amphipod fauna composition in a sector of the southern Gulf of Mexico (GoM). Previous records in the Gulf mainly comprised the oceanic province; our dataset included both oceanic and neritic zones, off several fluvial and lagoon systems. The biological material comprised 485 data records and a total abundance of 3,802 individuals.

**New information:**

Surveys were conducted at 21 sampling stations around the Veracruz Reef System National Marine Park, a marine protected area in the southern GoM. As a result of this research, we found 16 families, 34 genera and 78 species belonging to the suborder Hyperiidea. Our records include species from the oceanic province (up to 1,200 m depth), such as those from the genus *Scinia*, members of the infraorder Physosomata. In addition, *Lycaeopsiszamboangae* were found off the Alvarado Lagoon. Information on the habitat of 78 amphipod species (neritic, oceanic) is provided. The dataset is available at https://www.gbif.org/dataset/af18f3f8-f899-4c97-af47-8a110f856f92

## Introduction

Pelagic amphipods encompass a large number of crustacean species from the suborders Amphilochidea, Hyperiidea and Senticaudata, found worldwide. In tropical waters, however, researchers are still finding new records of species (Violante-Huerta et al. 2020, Domínguez-Nava et al. 2021, Violante-Huerta et al. 2021a) suggesting the need to improve the knowledge of their distribution around the world. Amongst the pelagic amphipods, the Hyperiidea is the dominant suborder with 292 species described in the world (Burridge et al. 2017). They exhibit greater diversity in the oceanic zone (Bowman and Gruner 1973, Lorz and Peracy 1975, Violante-Huerta 2019), yet various studies show that their abundance is higher in the neritic zone (Gasca 2004, Velázquez-Ramírez 2021). In the vertical plane, they occur from surface to abyssal depths, even in the hadal zone (Vinogradov et al. 1996, Vinogradov 1999). Hyperiids are carnivores and feed on other zooplankton organisms, such as polychaetes, chaetognaths, copepods, small crustaceans and even other amphipods (Bowman 1978, Williams and Robins 1981, Zeidler 1984). Furthermore, several species have been seen in association with gelatinous zooplankton, such as medusae, siphonophores and salps (Harbison et al. 1977, Laval 1980).

In the Gulf of Mexico (GoM), 17 families of the Hyperiidea suborder and a total of 119 species have been recorded (Gasca et al. 2009, LeCroy et al. 2009, Violante-Huerta 2019, Hereu et al. 2020). Hyperiid surveys in the GoM have mostly addressed the oceanic area and refer to new records of species, descriptions of intraspecific morphological variability and/or the analysis of some ecological features (Gasca 2003, Gasca 2004, Violante-Huerta 2019, Hereu et al. 2020, Violante-Huerta et al. 2021b). This study explores the composition of the hyperiid amphipod community in a sector of the southern Gulf that includes neritic and oceanic waters. This area is influenced by the discharge of freshwater outflows and contains the Veracruz Reef System National Marine Park, a protected natural area.

## General description

### Purpose

This study provides georeferenced information on the composition and abundance of hyperiid amphipods collected in the southern GoM during an oceanographic cruise.

## Project description

### Title

Pelagic amphipods (Crustacea: Amphipoda: Hyperiidea) from the southern Gulf of Mexico with notes on the distribution of species.

### Personnel

Laura Sanvicente-Añorve, Barbara Velázquez-Ramírez, Margarita Hermoso-Salazar.

### Study area description

**Figure 1.**
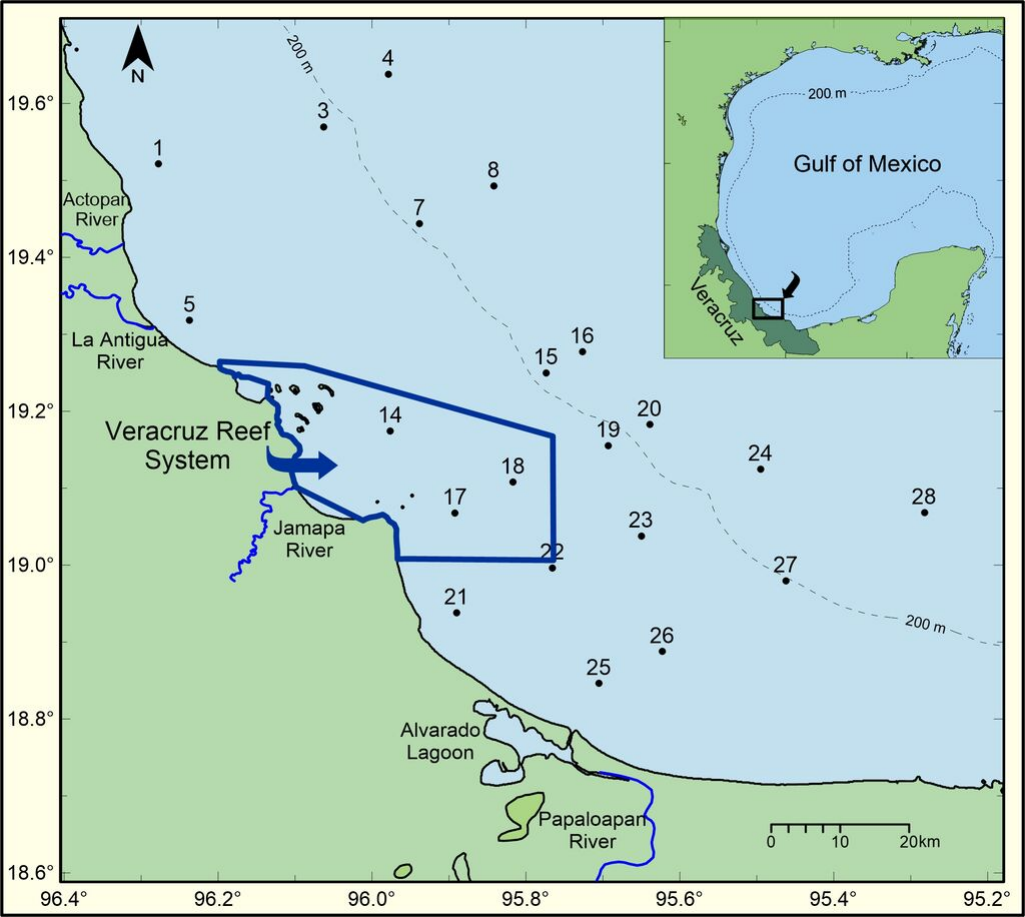
Study area and location of oceanographic stations.

The study area, located in the southern GoM, is included in Marine Ecoregion 14 of North America (Wilkinson et al. 2009). The sampling grid comprised neritic and oceanic zones (Fig. 1). The inner neritic zone is influenced by the discharges of several freshwater outflows, such as the Papaloapan River and Alvarado Lagoon. Surface circulation in the neritic zone exhibits a seasonal variation depending on the wind stress: during the autumn-winter period, circulation is towards the southeast, whereas during the spring-summer, surface waters flow towards the northwest (Zavala-Hidalgo et al. 2003). In the oceanic zone, the circulation pattern is cyclonic throughout the year (Pérez-Brunius et al. 2013, Sanvicente-Añorve et al. 2014).

### Design description

The dataset here provided included pelagic amphipod information from zooplankton collections performed at 21 sites in the southern Gulf of Mexico (Sanvicente-Añorve et al. 2022). Zooplankton samples were collected in the epipelagic region in both neritic and oceanic waters.

### Funding

Financial support came from the Instituto de Ciencias del Mar y Limnología, Universidad Nacional Autónoma de México.

## Sampling methods

### Study extent

The samples were obtained in the GoM between 18.84622 to 19.63814 North latitude and 95.28207 to 96.27735 West longitude, off the Mexican State of Veracruz. This area encompasses oceanic and neritic waters and contains the Veracruz Reef System National Marine Park, a protected natural area. The area is influenced by the discharge of the rivers Actopan, La Antigua, Jamapa, Papaloapan and the Alvarado Lagoon.

### Sampling description

Zooplankton samples were taken aboard the vessel “Justo Sierra”, during the oceanographic cruise named SAV-I, carried out from 29 November to 2 December 2007. The sampling grid comprised 21 neritic and oceanic oceanographic stations organised in six transects perpendicular to the coastline. Sampling was performed using a Bongo net of 333 and 505 µm mesh size; a flowmeter was placed at the mouth of each net to estimate the volume of filtered water. The net was towed for about 7 to 27 minutes (depending on bottom depth) at a speed of 2-3 knots. Collected samples were fixed in a 4% formaldehyde seawater solution buffered with sodium borate. Sampling depth ranged between 10 to 200 metres, depending on bottom depth.

### Quality control

Positions of sampling stations were georeferenced and displayed on a map using the Google Maps platform. The name of each species was verified using the taxon match tool of WoRMS (World Register of Marine Species).

### Step description

Amphipods were sorted from the samples collected with the 505 µm mesh size and preserved in 70% ethanol. The amphipods were identified, based on specialised literature (Stebbing 1888, Shih 1991, Vinogradov et al. 1996, Vinogradov 1999, Zeidler 1990, Zeidler 1992, Zeidler 1999, Zeidler 2000, Zeidler 2003, Zeidler 2004a, Zeidler 2004b, Zeidler 2016) and classified in accordance with Lowry and Myers (2017). Microdissections of taxonomically important structures (such as antennae, gnathopods, pereopods and uropods) were performed under the stereoscopic microscope to identify the hyperiids. The biological material is kept in the Laboratorio de Ecología de Sistemas Pelágicos of the Instituto de Ciencias del Mar y Limnología, Universidad Nacional Autónoma de México.

## Geographic coverage

### Description

The study area comprised neritic and oceanic waters off the Mexican State of Veracruz, in the southern GoM. Several freshwater outflows influence the area: the rivers Actopan, La Antigua, Jamapa, Papaloapan and the Alvarado Lagoon. A marine protected area, Veracruz Reef System, exists within the explored area (Fig. 1).

### Coordinates

18.84622 and 19.63814 Latitude; -96.27735 and -95.28207 Longitude.

## Taxonomic coverage

### Description

A total of 3,893 amphipods of the suborder Hyperiidea were sorted from the samples, from which 3,802 were identified to a species level. The identified individuals belonged to two infraorders, 16 families, 34 genera and 78 species (Tables 1, 2). Classification was after Lowry and Myers (2017).

Members of the Physocephalata infraorder, accounting for 99.4% of the total abundance, inhabit the epi- and mesopelagic layers and some species are considered characteristic of the tropical epipelagic fauna (Vinogradov et al. 1996, Vinogradov 1999). In contrast, species of the Physosomata infraorder (0.6%) are rare organisms that inhabit deep waters, but some species are frequently found in the upper epipelagic layer (Vinogradov et al. 1996, Gasca 2009). In this study, the Physosomata infraorder was represented by only one family, 10 species and 21 individuals, mostly found in the oceanic zone (Table 2).

The dominant species was *Lestrigonusbengalensis*, representing 67.7% (2,575 ind.) of all the identified hyperiids. It was found in all the surveyed sites, with the highest abundance in the neritic zone. This species has a circumtropical distribution in both neritic and oceanic waters, with its major abundance in shallow waters (Bowman 1973, Thurston 1976, Siegel-Causey 1982, Vinogradov et al. 1996).

Only seven additional species recorded an abundance percentage higher than 1%: *L.macrophthalmus* (163 ind.), *Lycaeopsiszamboangae* (115 ind.), *Eupronoeintermedia* (77 ind.), *Tetrathyrusforcipatus* (63 ind.), *Anchylomerablossevillei* (61 ind.), *Brachyscelusglobiceps* (55 ind.) and *Phronimopsisspinifera* (54 ind.). Except for the latter species, all these species were found in both oceanic and neritic areas (Table 2). In contrast, *P.spinifera* showed a clear tendency to occupy the oceanic zone. In particular, *L.zamboangae* was distributed in neritic and oceanic waters in front of the Alvarado Lagoon; previous studies indicated that the species has an affinity for surface waters of tropical and temperate regions (Siegel-Causey 1982, Zeidler 2004b).

Of the 78 species here recorded, only four were solely found in the neritic zone with one or two individuals: *Amphithyrussculpturatus*, *A.muratus*, *Schizoscelusornatus* and *Scinalepisma*. These species have been recorded in low numbers in oceanic areas (Gasca and Shih 2001, Gasca 2009, Violante-Huerta 2019, Hereu et al. 2020); thus, their presence in the neritic zone is not indicative of their main habitat.

## Temporal coverage

### Notes

From 29 November to 2 December 2007.

## Collection data

### Collection name

Zooplankton collection of the Laboratorio de Ecología de Sistemas Pelágicos from the Instituto de Ciencias del Mar y Limnología, Universidad Nacional Autónoma de México.

### Specimen preservation method

wet.

## Usage licence

### Usage licence

Creative Commons Public Domain Waiver (CC-Zero)

### IP rights notes

This work is licensed under a Creative Commons Attribution-NonCommercial-NoDerivatives 4.0 International Licence (CC BY-NC-ND 4.0).

## Data resources

### Data package title

Pelagic amphipods (Crustacea: Amphipoda: Hyperiidea) from the southern Gulf of Mexico.

### Resource link

https://www.gbif.org/dataset/af18f3f8-f899-4c97-af47-8a110f856f92

### Number of data sets

1

### Data set 1.

#### Data set name

Pelagic amphipods (Crustacea: Amphipoda: Hyperiidea) from the southern Gulf of Mexico.

#### Data format

Darwin core.

#### Download URL

https://ipt.iobis.org/caribbeanobis/resource?r=icml-lesp_pelagic_amphipods_s_gom

#### Description

This dataset contains georeferenced information of planktonic amphipods of the suborder Hyperiidea collected in front of the Mexican State of Veracruz, southern GoM, from 29 November to 2 December 2007. Zooplankton sampling was carried out with a Bongo net of 333 and 505 µm mesh size. The sampling grid included 21 oceanographic stations arranged in six transects perpendicular to the coastline. The study area is comprised between 18.84622 to 19.63814 North latitude and 95.28207 to 96.27735 West longitude. In total, the dataset includes 485 records corresponding to 78 species belonging to 34 genera and 16 families.

**Data set 1.** 

Column label	Column description
occurrenceID	An identifier for the Occurrence.
eventID	An identifier for the set of information associated with an Event.
stationName	An identifier for each oceanographic station.
eventDate	The date-time or interval during which an Event occurred.
minimumDepthInMetres	The lesser depth of a range of depth below the local surface, in metres.
maximumDepthInMetres	The greater depth of a range of depth below the local surface, in metres.
decimalLatitude	The geographic latitude (in decimal degrees, using the spatial reference system given in geodeticDatum) of the geographic centre of a Location.
decimalLongitude	The geographic longitude (in decimal degrees, using the spatial reference system given in geodeticDatum) of the geographic centre of a Location.
locality	The original textual description of the place.
country	The name of the country or major administrative unit in which the Location occurs.
countryCode	The standard code for the country in which the Location occurs.
geodeticDatum	The ellipsoid, geodetic datum or spatial reference system (SRS) upon which the geographic coordinates given in decimalLatitude and decimalLongitude are based.
coordinateUncertaintyInMetres	The horizontal distance (in metres) from the given decimalLatitude and decimalLongitude describing the smallest circle containing the whole of the Location.
samplingProtocol	The names of, references to, or descriptions of the methods or protocols used during an Event.
individualCount	The number of individuals present at the time of the Occurrence.
identifiedBy	A list (concatenated and separated) of names of people, groups or organisations who assigned the Taxon to the subject.
scientificName	The full scientific name.
scientificNameID	An identifier for the nomenclatural (not taxonomic) details of a scientific name.
scientificNameAuthorship	The authorship information for the scientificName formatted according to the conventions of the applicable nomenclaturalCode.
namePublishedIn	The four-digit year in which the scientificName was published.
kingdom	The full scientific name of the kingdom in which the taxon is classified.
phylum	The full scientific name of the phylum or division in which the taxon is classified.
class	The full scientific name of the class in which the taxon is classified.
order	The full scientific name of the order in which the taxon is classified.
family	The full scientific name of the family in which the taxon is classified.
genus	The full scientific name of the genus in which the taxon is classified.
specificEpithet	The name of the first or species epithet of the scientificName.
taxonRank	The taxonomic rank of the most specific name in the scientificName.
occurrenceStatus	A statement about the presence or absence of a Taxon at a Location.
basisOfRecord	The specific nature of the data record.

## Figures and Tables

**Table 1.** General taxonomic counts of hyperiid species.

**Infraorder**	**Families**	**Genera**	**Species**	**Records**
**Physocephalata**	15	32	68	466
**Physosomata**	1	2	10	19

**Table 2.** List of hyperiid species collected off Veracruz, southern Gulf of Mexico, in November-December 2007.

**Family/species**	**Number of individuals**	**Habitat of occurrence**
**Order Amphipoda Latreille, 1816** **Suborder Hyperiidea H. Milne Edwards, 1830** **Infraorder Physocephalata Bowman & Gruner, 1973** **Parvorder Physocephalatidira Bowman & Gruner, 1973** **Superfamily Phronimoidea Rafinesque, 1815** **Dairellidae Bovallius, 1887**		
*Dairellacalifornica* (Bovallius, 1885)	4	oceanic, continental slope
**Lestrigonidae Zeidler, 2004**		
*Hyperiettaluzoni* (Stebbing, 1888)	10	neritic, oceanic
*Hyperiettastebbingi* Bowman, 1973	16	neritic, oceanic
*Hyperiettastephenseni* Bowman, 1973	34	neritic, oceanic
*Hyperiettavosseleri* (Stebbing, 1904)	4	neritic, oceanic
*Hyperioideslongipes* Chevreux, 1900	31	neritic, oceanic
*Hyperionyxmacrodactylus* (Stephensen, 1924)	13	neritic, oceanic
*Lestrigonusbengalensis* Giles, 1888	2575	neritic, oceanic
*Lestrigonusmacrophthalmus* (Vosseler, 1901)	163	neritic, oceanic
*Lestrigonusschizogeneios* (Stebbing, 1888)	6	neritic, oceanic
*Lestrigonusshoemakeri* Bowman, 1973	1	oceanic
*Phronimopsisspinifera* Claus, 1879	54	oceanic
*Themistellafusca* (Dana, 1853)	35	neritic, oceanic
**Phronimidae Rafinesque, 1815**		
*Anchylomerablossevillei* H. Milne Edwards, 1830	61	neritic, oceanic
*Phronimaatlantica* Guérin-Méneville, 1836	4	oceanic, continental slope
*Phronimacolletti* Bovallius, 1887	3	oceanic
*Phronimacurvipes* Vosseler, 1901	11	neritic, oceanic
*Phronimapacifica* Streets, 1877	20	oceanic
*Phronimasedentaria* (Forskål, 1775)	2	oceanic
*Phronimasolitaria* Guérin-Méneville, 1844	1	oceanic
*Phronimastebbingi* Vosseler, 1901	10	oceanic, continental slope
**Phrosinidae Dana, 1852**		
*Phrosinasemilunata* Risso, 1822	7	neritic, oceanic
*Primnoabyssalis* (Bowman, 1968)	11	oceanic, continental slope
*Primnobrevidens* Bowman, 1978	7	oceanic
*Primnoevansi* Sheader, 1986	25	neritic, oceanic
*Primnojohnsoni* Bowman, 1978	8	oceanic, continental slope
*Primnolatreillei* Stebbing, 1888	9	oceanic, continental slope
**Superfamily Platysceloidea Spence Bate, 1862** **Amphithyridae Zeidler, 2016**		
*Amphithyrusbispinosus* Claus, 1879	9	oceanic
*Amphithyrusmuratus* Volkov, 1982	2	neritic
*Amphithyrussculpturatus* Claus, 1879	1	neritic
*Paralycaeagracilis* Claus, 1879	3	oceanic
*Paralycaeahoylei* Stebbing, 1888	1	oceanic
**Brachyscelidae Stephensen, 1923**		
*Brachysceluscrusculum* Spence Bate, 1861	4	neritic, oceanic
*Brachyscelusglobiceps* (Claus, 1879)	55	neritic, oceanic
*Brachyscelusrapacoides* Stephensen, 1925	17	neritic, oceanic
**Eupronoidae Zeidler, 2016**		
*Eupronoeintermedia* Stebbing, 1888	77	neritic, oceanic
*Eupronoemaculata* Claus, 1879	16	neritic, oceanic
*Eupronoeminuta* Claus, 1879	32	neritic, oceanic
*Parapronoecrustulum* Claus, 1879	1	continental slope
*Parapronoeparva* Claus, 1879	2	oceanic
**Lycaeidae Claus, 1879**		
*Lycaeapachypoda* (Claus, 1879)	13	neritic, oceanic
*Simorhynchotusantennarius* (Claus, 1871)	21	neritic, oceanic
**Lycaeopsidae Chevreux, 1913**		
*Lycaeopsisthemistoides* Claus, 1879	16	neritic, oceanic
*Lycaeopsiszamboangae* (Stebbing, 1888)	115	neritic, oceanic
**Oxycephalidae Dana, 1852**		
*Leptocotistenuirostris* (Claus, 1871)	12	oceanic, continental slope
*Oxycephalusclausi* Bovallius, 1887	1	oceanic
*Oxycephaluspiscator* H. Milne Edwards, 1830	9	neritic, oceanic
*Streetsiachallengeri* Stebbing, 1888	2	oceanic
*Streetsiaporcella* (Claus, 1879)	2	oceanic
*Streetsiasteenstrupi* (Bovallius, 1887)	1	oceanic
**Parascelidae Bovallius, 1887**		
*Schizoscelusornatus* Claus, 1879	2	neritic
*Parascelusedwardsi* Claus, 1879	21	neritic, oceanic
*Thyropussphaeroma* (Claus, 1879)	8	neritic, oceanic
**Platyscelidae Spence Bate, 1862**		
*Hemityphistenuimanus* Claus, 1879	13	neritic, oceanic
*Paratyphismaculatus* Claus, 1879	36	neritic, oceanic
*Paratyphisparvus* Claus, 1887	22	neritic, oceanic
*Paratyphispromontori* Stebbing, 1888	28	neritic, oceanic
*Platysceluscrustulatus* (Claus, 1879)	3	oceanic
*Platyscelusovoides* (Risso, 1816)	1	oceanic
*Platyscelusserratulus* Stebbing, 1888	4	oceanic
*Tetrathyrusforcipatus* Claus, 1879	63	neritic, oceanic
**Pronoidae Dana, 1852**		
*Pronoecapito* Guérin-Méneville, 1836	9	neritic, oceanic
**Superfamily Vibilioidea Dana, 1852** **Paraphronimidae Bovallius, 1887**		
*Paraphronimacrassipes* Claus, 1879	7	neritic, oceanic
*Paraphronimagracilis* Claus, 1879	5	oceanic
**Vibiliidae Dana, 1852**		
*Vibiliaaustralis* Stebbing, 1888	1	oceanic
*Vibiliapropinqua* Stebbing, 1888	2	oceanic
*Vibiliastebbingi* Behning & Woltereck, 1912	9	oceanic, continental slope
*Vibiliaviatrix* Bovallius, 1887	10	neritic, oceanic
**Infraorder Physosomata H. Milne Edwards, 1830** **Parvorder Physosomatidira Pirlot, 1929** **Superfamily Scinoidea Stebbing, 1888** **Scinidae Stebbing, 1888**		
*Acanthoscinaacanthodes* (Stebbing, 1895)	4	oceanic
*Scinaborealis* (G.O. Sars, 1883)	1	oceanic
*Scinacrassicornis* (Fabricius, 1775)	2	oceanic
*Scinaexcisa* Wagler, 1926	1	oceanic
*Scinalepisma* Chun, 1889	1	neritic
*Scinasimilis* Stebbing, 1895	1	oceanic
*Scinastenopus* Stebbing, 1895	5	oceanic
*Scinasubmarginata* Tattersall, 1906	3	oceanic
*Scinatullbergi* (Bovallius, 1885)	2	oceanic
*Scinavosseleri* Tattersall, 1906	1	oceanic
